# Dimensions of housing safety among women living with HIV in British Columbia: Results from a qualitative community-based research study

**DOI:** 10.17269/s41997-026-01167-0

**Published:** 2026-04-02

**Authors:** Madeline Gallard, Jennifer Demchuk, Leanne Zubowski, Joanna Mendell, Emily Taylor-Lariviere, Catherine Worthington

**Affiliations:** 1 Pacific AIDS Network (PAN), Vancouver, BC Canada; 2 School of Public Health and Social Policy, University of Victoria, Victoria, BC Canada

**Keywords:** HIV, Housing, Safety, Women, Community-based research, VIH, Logement, Sécurité, Femmes, Recherche communautaire

## Abstract

**Objectives:**

Housing plays a significant role in the health of people living with HIV. There are gender-specific and compounding effects of violence, trauma, and other structural factors that may interact with housing experiences for women living with HIV. We used a trauma-informed lens to explore how people who identified as women (i.e., cisgender and transgender women) living with HIV in British Columbia perceived housing safety, and strategies they employed to manage or address threats to housing safety.

**Methods:**

A sub-sample of 49 participants who identified as women was selected from a larger community-based research project, *Positive Living, Positive Homes*, a longitudinal qualitative comparative case study of housing experiences among people living with HIV. Local coordinators conducted semi-structured interviews at two time points, approximately 12 months apart. Interviews were recorded and transcribed for descriptive thematic analysis.

**Results:**

We identified three inter-related dimensions of safety in housing for women living with HIV: physical, psychological, and financial safety. These safety themes were consistent across different cultural identities, although African immigrant women faced distinct financial safety pressures. Narratives around housing safety revealed the importance of other identities (e.g., as parents or caregivers) in addition to being women living with HIV. Within their housing situations, participants were continuously building safety mechanisms, and many experienced cycles of safety and lack of safety.

**Conclusion:**

To recognize the safety needs and trauma histories of many women living with HIV, gender-, trauma-, and violence-informed approaches to housing and housing services are needed.

## Résumé

**Objectifs:**

Le logement joue un grand rôle dans la santé des personnes vivant avec le VIH. La violence, les traumatismes et d’autres facteurs structurels ont des effets sexospécifiques et conjugués qui peuvent interagir avec les expériences de logement des femmes vivant avec le VIH. À l’aide d’un prisme sensible aux traumatismes, nous avons exploré comment des personnes s’identifiant comme femmes (c.-à-d. les femmes cisgenres et transgenres) et vivant avec le VIH en Colombie-Britannique perçoivent la sécurité du logement, et quelles sont les stratégies qu’elles emploient pour gérer ou aborder les menaces à la sécurité du logement.

**Méthodes:**

Nous avons sélectionné un sous-échantillon de 49 personnes s’identifiant comme femmes dans le cadre d’un projet de recherche communautaire plus vaste, *Positive Living, Positive Homes*, une étude de cas longitudinale qualitative qui compare les expériences de personnes vivant avec le VIH en matière de logement. Une équipe de coordination locale a mené des entretiens semi-directifs à deux dates différentes, à environ 12 mois d’intervalle. Les entretiens ont été enregistrés et transcrits pour les besoins d’une analyse thématique descriptive.

**Résultats:**

Nous avons cerné trois dimensions interdépendantes de la sécurité du logement pour les femmes vivant avec le VIH: la sécurité physique, psychologique et financière. Ces trois aspects étaient constants quelle que soit l’identité culturelle, mais les immigrantes africaines subissaient des pressions particulières sur le plan de la sécurité financière. Les descriptions de la sécurité du logement ont révélé l’importance d’autres identités (p. ex. celle de parent ou de proche aidante) en plus de l’identité de femme vivant avec le VIH. Dans leur situation de logement, les participantes établissaient continuellement des mécanismes de sécurité, et beaucoup vivaient des cycles de sécurité et d’insécurité.

**Conclusion:**

Pour avoir conscience des besoins de sécurité et des antécédents de traumatismes de nombreuses femmes vivant avec le VIH, il faut aborder le logement et les services de logement selon des approches sensibles au genre, aux traumatismes et à la violence.

## Introduction

Living with human immunodeficiency virus (HIV) poses distinct challenges for women. Approximately one-quarter (25.3%) of people living with HIV in Canada are female, estimated to be 16,544 females in 2022 (Public Health Agency of Canada (PHAC), 2024). In 2022, 656 (35.5%) of new HIV infections were in females, and although ethnicity data are inconsistently reported, new cases were disproportionately among Indigenous, African, Caribbean, and Black women (PHAC, 2024). While information for transgender women living with HIV in Canada is not available, worldwide, trans women have a higher HIV prevalence than trans men and the general population (Stutterheim et al., 2021). In addition to increased biological susceptibility for females, structural and social factors such as gender norms and caregiving roles, poverty, housing, racism and discrimination, gender-based violence, and the legacies of colonization increase women’s risk for acquiring HIV (Gahagan & Dworkin, 2021). Women living with HIV in Canada have lower diagnosis (86% vs 90%), treatment (84% vs 86%), and viral suppression (92% vs 96%), and experience poorer health outcomes than men living with HIV (Loutfy et al., 2021; PHAC, 2024) .

Housing is a key intermediate social determinant of health. People who are unstably housed or live in inferior housing situations are at increased risk of HIV transmission and have associated poorer treatment access and adherence (Aidala et al., 2016; Reddon et al., 2023; Zhao et al., 2023). Greene and colleagues (2010) discuss obstacles in accessing suitable housing for parents (predominantly women) living with HIV and their children, such as HIV-related stigma and discrimination, including being pressured to move if others found out their HIV status. A study with 336 women living and accessing HIV care in Vancouver, British Columbia (BC), found the majority (71%) had experienced unsheltered or unstable living conditions in the previous 6 months, with key associations found between unstable housing (compared to stable) to living in the Downtown Eastside (a neighbourhood characterized by high levels of poverty and drug use), hospitalization, and physical and/or sexual violence in the last 6 months (Zhao et al., 2022).

The impact of lifetime experiences of violence, including gender-based violence, is a significant concern, as there are high rates of lifetime violence among women living with HIV. A 2014 study (Borwein et al., 2014) reported that over 80% of 249 women living with HIV had experienced violence in their lives, and a more recent study of cis- and transgender women living with HIV in Vancouver (Ohtsuka et al., 2023) found that 96% of participants had experienced physical and/or sexual violence during their lifetime. These experiences of violence often intersect with stigma and depression, drug use, incarceration, and/or sex work (El-Bassel et al., 2022; Logie et al., 2019; Ohtsuka et al., 2023). A qualitative study with Indigenous women living with HIV in Canada revealed a significant role of experiences of violence in HIV risk, including emotional, sexual, and physical abuse, often starting in childhood, and based in the legacies of colonization, including intergenerational harms of residential schools (Hawkins et al., 2009).

A trauma-informed approach recognizes how trauma, “the lasting emotional response that often results from living through a distressing event” (Centre for Addiction and Mental Health (CAMH, n.d.), intersects with health and social problems people face when seeking help. This approach aims to address trauma sensitively together with a person’s other issues, using six core principles of safety, trustworthiness, collaboration, empowerment, choice, and intersectionality, and acknowledges oppressive practices of systems and structures (Bowen & Murshid, 2016). Trauma and violence aware care have been promoted as foundational elements within a Canadian Women-Centred HIV Care Model, to facilitate a sense of control and empowerment in the healthcare environment (Loutfy et al., 2021).

### Study purpose

Set within a market, policies, and regulations, housing is a physical space where one can experience safety (or risk/danger), and a desired psychological space of belonging or “home” (Handel, 2019). Housing plays a significant role in the health of people living with HIV, and there are gender-specific and compounding effects of violence, trauma, and other structural factors that may interact with housing experiences for women living with HIV. As part of a larger community-based, qualitative comparative case study on housing for people living with HIV in three BC communities, we used a trauma-informed lens to explore how people who identified as women (i.e., cisgender and transgender women) living with HIV perceived dimensions of safety in their experiences of housing situations (inclusive of unhoused to market housing), any differences across social location (community, age, ethnicity), and strategies they employed to manage or address threats to housing safety.

## Methodology and methods

### Study design

The *Positive Living, Positive Homes* study used a community-based research (CBR) approach to conduct a longitudinal qualitative comparative case study of housing experiences among people living with HIV. CBR values the expertise and knowledge of people with lived and living experience, the complex and multiple understandings of lived reality set within health inequities and social and structural determinants and constraints, and through power sharing promotes co-learning and community empowerment (Wallerstein et al., 2018). Our embedded comparative case study methodology allowed us to use our primary unit of analysis—the experiences of people living with HIV over time as they navigated housing—and examine it within three case sites (communities) nested within the BC temporal, geographic, and structural contexts (Yin, 2009).

*Positive Living, Positive Homes* was co-led by the last author and PAN, a BC HIV, hepatitis C, harm reduction, and community services network. Fourteen people living with HIV, including Peer Consultants and three Peer Research Associates, were involved through the phases of the research over an 8-year period, including research question and funding proposal development, interview schedule development, recruitment, data analysis and interpretation, knowledge translation, and community product and paper development. Our team included two Indigenous people living with HIV and three representatives from Indigenous and African immigrant organizations. Ethics approval was received from the University of Victoria Human Research Ethics Board (14–182).

### Setting and recruitment

Three communities in different health regions in BC participated in *Positive Living, Positive Homes*: a large urban centre (Community 1) and two smaller centres (Communities 2 and 3). Local study coordinators and Peer Research Associates were engaged from each centre to be part of recruitment, interview guide development, and data interpretation. People living with HIV in these three communities were recruited to be participants through passive methods, including posters and e-mail through mailing lists sent by community agencies. Participants were selected using purposive sampling to maximize variability in living situation (from unhoused to market housing) and personal characteristics (e.g., gender, sexual orientation, ethnicity, family structure, time living with HIV). Local study coordinators contacted study participants every 3 to 4 months to enable follow-up interviews one year later.

### Methods

Study methods included two semi-structured interviews (baseline and follow-up), a timeline exercise, and an arts-based mapping technique. The interview guides were designed to allow participants to reflect on their experiences of housing and health. The first guide included questions about amenities of the participant’s housing environment (e.g., counter space, a shower) and any concerns they had about the home, particularly elements that influenced health and wellbeing. During this baseline interview, participants were asked to complete a timeline exercise that outlined their housing history (for 5 years prior). They were also invited to participate in an arts-based technique that allowed them to share lived experiences more deeply without the dependence on oral expression (Bagnoli, 2009). This method included drawing a “map” that described their current living situation and things, using green for enablers of health and red for barriers to health. The interview guide for the second interview referred to the timeline created in the first interview, using it to discuss the participant’s housing experience over time and allowing for a holistic view of the interaction of housing and living with HIV.

### Data generation

Local study coordinators conducted the interviews in-person at two time points (2015–2016), with the second interview occurring approximately 12 months after the first. The interviews were recorded and transcribed for analysis. The baseline interviews lasted approximately 2 h; follow-up interviews lasted approximately 1 h. Interviews were held within community-based organizations. At the request of participants, eight interviews included two participants. Participants were compensated $25 for each interview.

### Data analysis

The CBR team completed descriptive thematic analysis of all transcripts, and community gatherings were held to interpret themes in each of the participating communities. It became apparent during the preliminary analysis that “choice/autonomy” and “things that create/do not create safety” were prominent themes, particularly for women participants. A smaller team who was interested in this issue then conducted more extensive analysis of the data from the 49 participants who identified as women (i.e., cisgender and transgender women) around these themes, including participant descriptions of their housing maps. (More in-depth analyses (e.g., (Mitchell et al., 2011) of drawings are not included in these results.)

The six team members who conducted this analysis identified as women or nonbinary, and some had experienced gender-based violence. All identified as settler-colonists. Check-ins with people living with HIV (including white, Indigenous, and women of colour) were held during the analysis process to receive feedback and augment interpretation.

In this sub-analysis, we used a trauma-informed lens to focus our attention on social and environmental contexts of women who have experienced violence and to position women who have experienced violence and trauma as having choice and strengths. Thus, our aim was to analyse the intersections of trauma and violence with the ways women participants experience safety/lack of safety within housing, and their strategies for managing threats to safety. Further, we were attentive to the ways in which social location (community, age, ethnicity) might impact these experiences.

## Results

### Participant characteristics

In total, 99 people living with HIV completed a baseline interview, and 71 (72%) completed a follow-up interview. For this analysis, we focus on the sub-sample of 49 participants who identified as women (27 in Community 1, 6 in Community 2, and 16 in Community 3), 36 of whom completed follow-up interviews. Three participants identified as transgender women. The majority of participants (*n* = 31) identified as Indigenous (either First Nations or Metis); 10 as white, and 6 as African immigrant. Ages ranged from 30 to 68 at the time of the first interview (median age 46). The majority (*n* = 31) reported living in market housing at time 1, with 6 in non-profit or geared-to-income housing, and 4 in a shelter or homeless. Although they were not asked directly about experiences of violence, just under half (*n* = 22) of the women described physical or sexual violence and abuse during their lifetimes in the first or second interview.

### Safety for women living with HIV

We identified three key dimensions of safety as described by the participants: physical safety, psychological safety, and financial safety. These dimensions were often interconnected in participant narratives. For example, one participant marked in red (barrier to health) on her housing map an area of a transitional housing building where fights occurred, and discussed how this area created physical (“Guys … want to fight people”) and psychological (“I don’t feel safe”) threats to safety (Indigenous woman, 44 years old, Community 2).

#### Physical safety

Physical conditions of housing, such as mold, exposure to heat or cold, and unmaintained elements of housing that put women at health risk were described extensively by participants. One woman described flooding after the toilet was flushed:

Water [was an issue]. I will have an appointment [to go to]; then I – that morning after everything [I did away from housing] … I go look: my room, filled with water.

(African woman, 59 years old, Community 1)

Another participant described pollution from a leaking oil tank:

Yeah, furnace oil, cause it, I guess the trailer [I lived in] used to be heated like that way, and then he [former roommate] forgot to empty out the tank and so he kind of polluted all the grounds … So, I was sitting in a room huffing gas pretty much for, I don’t know, eight months straight.

(Indigenous woman, 40 years old, Community 3).

Another participant stated, “Well, a couple of the places had mold. And I didn’t think it would be good for me because I’m HIV [positive].”

(Indigenous woman, 48 years old, Community 1).

Several participants referenced pests on their maps, showing a physical threat to their safety. For example, a participant marked in green (health promoting element) a room that did not have carpet and indicated the lack of carpet made her feel better because she had struggled with bedbugs previously. Discussions around pests included concerns that the pests would return and strategies to prevent this, as one participant described while discussing a point of entry for mice:

Mind you, my cats nipped that in the bud pretty quick, so I haven’t seen any. But now they’re … you can tell they’re doing their little stance - so they’re smelling [for mice], right. So … now I got two kittens, too …

(Indigenous woman, 48 years old, Community 1)

Some participants described physical elements of the living environment as putting them at risk for violence. One woman, who was experiencing homelessness and camping on the street, included in her map an area labelled in red to indicate exposure of her camp to threats, noting, “The entire thing is open, for anybody to walk into … On this side of the fence, drug dealing. I can’t draw trees, but these trees are full of animals. Raccoons, and skunks. (white/Métis woman, 48 years old, Community 1). Another woman described a sense of threat of physical attack coming from big windows, “I like the big windows for during the day, but not at night. They scare the crap out of me. And that’s just from past trauma.” (Indigenous woman, 44 years old, Community 2).

Many participants viewed security measures, such as security staff, cameras, and door locks, as important for and supportive of their concept of safety and security.

Interviewer: Do you feel secure and stable in your housing situation?

Participant: Yes.

Interviewer: Why is that?

Participant: They … it’s kind of like … like a block watch. So, everybody upstairs keeps an eye out. Nobody’s allowed to let anybody in that doesn’t have a key. Like, we have a fob and if there’s somebody out there, you don’t let the person in, you let them buzz.

(Indigenous woman, 27 years old, Community 1)

Participants who were parents or caregivers reported safety considerations for housing as it related to children within their home. One participant noted the deck area of her home was not safe for children:

The spindles aren’t gapped to code, where they’re supposed to be four inches apart. And I noticed that because I babysat... they were sticking their heads through.

(white woman, 37 years old, Community 1).

Further, some participants noted that their own health related to HIV and other health challenges impacted their physical ability to maintain a home to keep it clean and safe; this was noted as an area where some participants could use support (e.g., housekeeping services), particularly as they aged. One participant explained:

Now it’s hard for me to keep house. It’s hard for me to bend down and clean my bathroom. It’s … I can sweep my floor, but then I have to rest before I can vacuum.

(Indigenous woman, 44 years old, Community 1).

The ways in which the participants describe physical safety in their home related to elements that inhibit violence and pests, as well as elements that promote health and safety for children. Participants also discussed how physical safety can be supported, including security measures like locks on doors, and services that support them, given physical health limitations, to keep their houses clean and safe.

#### Psychological safety

Descriptions of psychological safety described by participants were predominantly about safety from intimate partner violence and other forms of violence and abuse. Safety within family and other social relationships was also a consideration for some participants; three participants marked in red areas of the home where family members caused tension or stress (e.g., a couch that a particular family member often spent time on).

Some participants made only passing mention of experiences of violence; for example, one participant mentioned childhood experiences of violence while describing the layout of her living space. However, many participants described experiences of violence and abuse across their lives, from childhood through adulthood. As one woman explained:

So, I had a way of life [experiences of violence] that I was used to living, so I did have to work on a lot of issues... I had to work with, like … anti-violence issues, or violence from my past.

(Indigenous woman, 48 years old, Community 1).

In terms of housing and living spaces, participants made intentional self-management choices to increase their safety, considering the risk of violence and abuse. For example, one participant described altering her walking route through her neighbourhood to avoid harassment:

I cross the road to walk on the opposite side of the street because I can’t stand walking by their building and getting whistled at or called, and if I don’t respond, getting called a “fat cow”… like, it’s just ridiculous.

(white woman, 48 years old, Community 1)

Another participant described a strategy for her exit from an abusive relationship:

I might even move right out of [city] so then, that way, I don’t have to see him at all. But it’s expensive. I just won’t let him know where I’m living, and I’ll tell them here not to give out my address.

(white woman, 54 years old, Community 1)

In some cases, participants noted that these strategies resulted in their considering and making trade-offs around safety, as one participant explained when describing changing neighbourhoods:

Participant: To tell the truth, I left an abusive relationship last year, so I gave my [housing] notice and I left, and I was homeless for six months. … I was going to be on the streets for the winter.

(Indigenous woman, 64 years old, Community 1)

The ways in which participants discussed psychological safety was closely intertwined with physical safety. Women discussed how abuse and impacts of intimate partner violence, as well as tensions with family members, interacted with their use of living spaces. As one woman described:

Participant: He never wanted me to see a doctor or access care …

Interviewer: So, your mental health medications, you had to hide it with a neighbour? 

Participant: Mm-hm.

(African woman, 41 years old, Community 1).

Some participants also described experiencing sexual harassment from their landlords or other people living in their shared space and witnessing violence. As one woman explained,

There’s a couple of times, like when I first moved in, the landlord kind of came onto me because he drinks. Yeah. And then he attacked his wife and asked me to hide the weapon. And yeah, it was kind of – put me in bad situations, right.

(white woman, 54 years old, Community 1).

The strategies women used to mitigate threats to psychological safety included avoidance as well as trade-offs between elements of physical and psychological housing safety, including moving away from abuse; however, this could compromise housing stability. Results demonstrated how housing situations and experiences of violence produce threats to psychological wellness, as well as threats to physical health.

#### Financial safety

Many participants expressed that their financial ability to continue to be able to live in one place was very important for a sense of safety and security. As one participant summarized, “Security – I guess it would mean that I’d be able to live there as long as I need to.” (Indigenous woman, 32 years old, Community 1). Many participants described challenges securing housing they perceived as safe at a manageable cost and described worries about other costs impacting their overall safety. While most participants spoke of financial safety, there were distinct financial safety issues for African immigrant women, as one woman explained:

You see, because in [immigrant transitional housing] people - lots of people - can only stay fifteen days. And when you stay your fifteen days, the money they’re going to give you, they will cut it and you will have to pay for the place.

(African woman, 39 years old, Community 1).

A second African immigrant participant described struggles with receiving a return on a deposit from a landlord: “I say, ‘I’m not moving here,’ and he say, ‘If you don’t want to move, you’re going to lose your deposit.’” (African woman, 43 years old, Community 1).

Participants described financial stabilization as an important supportive factor in their sense of safety and security within their homes. As one woman explained, “I know my rent is paid and I don’t have to worry about my power being cut off or anything. And yeah, I don’t have any worries.” (Indigenous woman, 47 years old, Community 1). In one participant’s map, she deepened the shade of green in the drawing and described that the space felt better than before as she had more ownership over the space, having started a home-based business. A second participant described feeling positively about receiving a portable housing subsidy, as this increased her housing mobility and allowed her to live alone.

Participants had a range of strategies to promote financial security and safety in housing, including having illegal sub-tenants:

Yeah, I have [a] roommate. I rent out my spare bedroom, which is under the radar... I get the rent paid in cash rather than a cheque, so that it’s an undeclared income.

(white woman, 48 years old, Community 1)

In addition, several women indicated that they did not call attention to the need for repairs out of concern it would induce a rent increase, as described by one woman:

When you ask to get certain things fixed or for improvements, it’s kind of like a balance because I don’t want to – I don’t want to rock the boat in the sense of putting – for getting high rent. I don’t know how the owner looks upon, “Oh well we fixed this, this and this, so maybe we can put her rent up.”

(white woman, 46 years old, Community 1)

An example of reflecting on self-management choices surrounding financial safety is summarized by a participant and her male partner who described regrets about disclosing their relationship as it impacted social assistance:

Interviewer: … For your … social assistance … do you … list that you’re a couple or do you not disclose to them that you’re together?

Participant: He added me on his.

Male partner: Yeah, we should’ve kept it separate because we would’ve gotten a little more [social assistance].

(Indigenous woman, 40 years old, Community 3)

For women in the study who were parents or caregivers, concerns around financial security and safety tied into concerns around space for children. One participant described sharing a bed with her grandchild, as she did not have the space in her apartment for a second bed. Another participant noted that she slept in the living room so each of her teenage children had their own bedrooms.

Legal policies surrounding children and custody agreements also impacted financial safety for participants who were mothers. One participant noted that government policies surrounding the number of bedrooms that must be available to accommodate her family created challenges, and another discussed weighing the costs of living in different communities to accommodate custody agreements’ limitations on when she could spend time with her children.

Participants discussed how financial security led to housing stability and increased feelings of safety. Women discussed the interactions between finances and stability with regards to legal obligations in child custody agreements, strategies for financial management, and how these factors could be in conflict and compromise their stability and safety. Participants also discussed avoiding advocating for improvements to housing in case it threatened their housing stability, and financial implications of living with a partner or roommate.

## Discussion

Working from a trauma-informed approach, this analysis revealed that the women living with HIV in this study experienced safety in housing within three inter-related dimensions: physical, psychological, and financial safety. Just under half spontaneously disclosed that they had a history of experiencing violence and abuse. Across the three sites, the majority of participants (31) self-identified as Indigenous; our sample also included 6 women who were immigrants from Africa. Despite cultural variability, themes were consistent across participants from differing cultural identities. An exception was the distinct financial safety pressures faced by African immigrant women around settlement and income. Such pressures were also noted in other research on migrant women in Canada (Hanley et al., 2019).

Women living with HIV developed strategies to manage their housing experiences related to lack of safety by avoiding or leaving situations of violence. The analysis also suggested that these women are skilled at developing management strategies to enhance their housing safety. A qualitative study of trauma among people living in a BC HIV housing facility similarly found that participants made deliberate choices to protect their safety (e.g., self-isolation from interaction with others in the building to avoid retraumatization) when they felt there was a threat to their safety (Koehn et al., 2020). Assessing housing safety in the context of other choices related to safety often involved making complex trade-offs, such as moving out of secure housing to leave an abusive relationship; this finding was similar to Greene and colleagues’ (2010) study of parents (predominantly women) living with HIV.

Concerns about safety in this study revealed the intersection of participants’ identities beyond being women living with HIV, especially their roles as parents or caregivers and their physical and financial abilities to maintain a safe home environment. Many of our study participants emphasized the importance of physical security measures like security staff, cameras, and locks in enhancing their sense of safety. This finding is similar to findings from the HIV housing facility study (Koehn et al., 2020), but contrasts with another study that found security measures like cameras could reduce safety, increase vulnerability, and create a sense of surveillance for women using drugs (Collins et al., 2018). This contrast illustrates the importance of context and approaching support services from an intersectionality perspective (McCall, 2005).

Women in our study considered their housing situations within the context of setting or neighbourhood. This included a lack of physical and psychological safety for women who were unhoused (e.g., camping); others also felt threatened in their neighbourhood (e.g., harassment from people at a neighbouring building). A study of men and women living with HIV in BC found that women perceive significantly less neighbourhood cohesion (e.g., social connection to neighbours, psychological sense of community) than men, with their sense of neighbourhood cohesion being positively associated with stable housing (Closson et al., 2018).

Our study participants spoke to their own lived experiences set within the housing markets, policies, and programs in BC. Many of the factors promoting or inhibiting their safety are issues that need to be addressed at the structural level: safety codes and regulations impacting housing stability, quality, rent levels, income support, and adaptations for ageing and disability. In interacting with these systems, our participants were continuously building their own safety mechanisms, and many experienced cycles of safety and lack of safety, such as when housing became too expensive and forced a move, or the form of housing became untenable with age-related changes in ability. Handel (2019) describes this process as, “a performative act intended to reconstruct the damaged, imperfect home into a safe place it should have been” (p. 1052).

Supportive housing for people living with HIV is an essential part of the continuum of HIV care and it is important to include considerations of gender-, trauma-, and violence-informed care (Zhao et al., 2022). Applying a trauma-informed approach to housing and housing services has been shown to improve the housing experience for those with trauma histories. Bebout (2001) suggests using this approach can help clients develop new self-management and relationship skills, making them feel more connected to their homes. Koehn and colleagues (2020) emphasize the importance of understanding how housing interventions interact with trauma histories. They also suggest that a trauma-informed approach can benefit training, policies, and practices for supportive housing staff. Bebout (2001) suggests practical ways for housing providers to incorporate a trauma-informed approach in policies and practices, such as considering trauma experiences when assessing housing needs, giving clients more housing choices, and respecting their privacy and boundaries by providing private bedrooms and privacy locks. There are also emerging approaches to understanding the built environment (e.g., a housing unit) as places where community wellbeing is developed within shared spaces through collective action; thus, the built environment and building design becomes an important tool to promote community wellbeing, beyond dimensions of safety and security (Lach et al., 2022).

### Strengths and limitations

Qualitative research can provide rich information about select experiences set within a specific context. Our results speak to the experiences of 49 women living with HIV in three communities in BC, set within BC’s policy and social contexts. Our results provide rich information about the women’s experiences of safety (or lack thereof). We worked to include people from populations most impacted by HIV, and so our sample includes Indigenous women, as well as African immigrant and white women. Our focus on housing meant that we did not ask questions related to family history and intergenerational trauma, or the comprehensive immigrant experience. Further study with women from these populations using Indigenous and/or anti-racist approaches is clearly warranted. It should also be noted that these interviews were conducted prior to COVID-19. Research suggests that COVID-19 lockdown periods exacerbated violence against women (The Lancet Public Health, 2022); thus, it is likely that stress and safety concerns among women living with HIV around housing would have increased during this period.

Trauma-informed perspectives in considering housing for women living with HIV must be informed by the understanding that trauma experiences and their impacts do not sit solely within an individual; rather, they are indicative of wider structural issues and point to trauma being rooted in the social determinants of health (McKenzie-Mohr & Lafrance, 2017). Systems health equity and intersectional perspectives recognize that these experiences tie to intersections of lived experiences (e.g., gender, family status, and substance use) and social determinants of health, such as poverty, colonization, access to housing, and childhood experiences (McCall, 2005; Pauly et al., 2018). These lifetime experiences can contextualize elements of the physical home and become crucially important in considerations of what makes housing safe, as well as how safety contributes to overall health equity, and how supportive services can be organized to support diverse identities. It is important that interventions move beyond focusing on an individual level to affect change at the structural level to better support health equity and acknowledge that threats to safety are intrinsically linked to marginalization and structural systems of inequity, such as patriarchy, HIV-related stigma, and discrimination based on family status.

## Contributions to knowledge

What does this study add to existing knowledge?

- This study uses a trauma-informed approach to identify dimensions of safety that affect the experiences of accessing and maintaining housing for women living with HIV and strategies women employ to manage safety.
- Women living with HIV experience unique considerations for safety, including gendered considerations such as safety from violence and maintaining the safety of children, establishing safety with histories of complex trauma and violence, and finding housing that meets the needs of an individual living with HIV.

What are the key implications for public health interventions, practice, or policy?

- Public health policy in relation to housing for women living with HIV must recognize that these women have complex understandings of safety and are capable of weighing the “trade-offs” required to promote their own safety.
- Building a trauma-informed approach into housing policy for women living with HIV requires recognizing the safety needs and trauma histories of many women living with HIV, while respecting and promoting their self-developed safety pathways.

## Data Availability

Text data files are confidential. Supplemental reports are available on the PAN website: https://paninbc.ca/research-and-evaluation/plph/
